# Genomic surveillance reveals geographical heterogeneity and differences in known and novel insecticide resistance mechanisms in *Anopheles arabiensis* across Kenya

**DOI:** 10.1186/s12864-025-11788-3

**Published:** 2025-07-01

**Authors:** Brian Polo, Kelly L. Bennett, Sonia Barasa, Jon Brenas, Silas Agumba, Joseph Mwangangi, Lucy Wachira, Stanley Kitur, Damaris Matoke-Muhia, David M. Mburu, Edith Ramaita, Elijah O. Juma, Charles Mbogo, Eric Ochomo, Chris S. Clarkson, Alistair Miles, Luna Kamau

**Affiliations:** 1 Centre for Global Health Research (CGHR), Kenya Medical Research Institute, Kisumu, Kenya; 2 Vector Genomic Surveillance Unit, Wellcome Trust Sanger Institute, Hinxton, Cambridge, UK; 3 Pan-Africa Mosquito Control Association (PAMCA), Nairobi, Kenya; 4 Centre for Geographic Medicine Research-Coast (CGMR-C), Kenya Medical Research Institute, Kilifi, Kenya; 5 Center for Biotechnology Research and Development (CBRD), Kenya Medical Research institute, Nairobi, Kenya; 6 Pwani University Biosciences Research Centre (PUBReC), Kilifi, Kenya; 7 Ministry of Health - National Malaria Control Programme (NMCP), Nairobi, Kenya; 8 Centre for Infectious and Parasitic Diseases Control Research, Kenya Medical Research Institute, Busia, Kenya; 9 Liverpool School of Tropical Medicine, Liverpool, UK

**Keywords:** Insecticide Resistance, *Anopheles arabiensis*, Population Genetics, Malaria vector, Kenya

## Abstract

**Background:**

Insecticide resistance in disease vectors poses a significant threat to the control of transmission globally. In *Anopheles* mosquitoes, resistance has jeopardized gains made in malaria control and led to the resurgence of cases. Although *Anopheles arabiensis* is a major malaria vector, little is known about its genetic diversity and insecticide resistance mechanisms across geographical space. There is an urgent need to incorporate genomics in resistance monitoring to allow preemptive detection of adaptive alleles.

**Methods:**

We analyzed whole-genome data from 498 *An. arabiensis* specimens collected across five regions in Kenya. Population structure was assessed and both known and novel resistance mechanisms were investigated through SNP and CNV frequency analysis, genome-wide selection scans and haplotype clustering.

**Results:**

Analyses of whole-genome data revealed geographical population structure between the northwestern region and central coastal Kenya, which was likely influenced by the Great Rift Valley. Distinct geographical differences in insecticide resistance profiles were observed across Kenya, reflecting differences in ecology, land use and selection pressure. For instance, in central Kenya, copy number variants at the Cyp6aa/p gene cluster and carboxylesterase genes associated with metabolic resistance to pyrethroids and organophosphates are fixed. In contrast, northwestern Kenya had mutations associated with both the target site and metabolic resistance to pyrethroids and DDT at high frequencies. Vgsc-L995F mutations occurred at frequencies of up to 44%, and duplications of *Cyp9k1* occurred at frequencies of up to 66%. Genome-wide selection scans identified novel candidates under selection in central Kenya, including the Keap1 gene, which is involved in the regulation of multiple detoxification genes, likely due to high insecticide pressure in the region.

**Conclusion:**

Restricted gene flow coupled with heterogeneity in molecular insecticide resistance across Kenya suggests that localized control measures may be more effective in preventing the spread of insecticide resistance in *An. arabiensis*. This study highlights the importance of incorporating genomics in the routine monitoring of malaria vector populations to identify the emergence of new resistance signatures and their geographic distribution and spread.

**Supplementary Information:**

The online version contains supplementary material available at 10.1186/s12864-025-11788-3.

## Introduction

The emergence of insecticide resistance in malaria vectors poses a formidable threat to global malaria eradication [1]. This is despite significant advancements in global malaria control through the widespread use of insecticide-based interventions such as insecticide-treated nets (ITNs) and indoor residual spraying (IRS) [2]. An increase in insecticide resistance has hindered progress toward the World Health Organization’s (WHO) Global Technical Strategy for malaria elimination and exacerbated the resurgence of the disease [3]. Recently, the number of global malaria cases has increased from 229 million in 2019 to 263 million in 2023, with the WHO African Region accounting for 93.5% of these cases [4].

Geographic and ecological architecture significantly impacts malaria vector population dynamics and plays a crucial role in shaping the prevalence and distribution of insecticide resistance mechanisms [5]. For example, geographical or environmental barriers may restrict gene flow and therefore the sharing of adaptive alleles [6, 7]. Kenya’s diverse landscape, characterized by distinct climatic zones, influences the prevalence and distribution of malaria vectors and their associated resistance patterns [8]. Ecological conditions range from arid and dry in the north to hot and humid along the coast, with the central highlands experiencing a cooler climate [9]. Understanding how insecticide resistance mechanisms are distributed across this heterogeneous landscape is crucial for developing targeted and effective vector control strategies.

Insecticide-treated nets (ITNs) are used as the country’s primary malaria vector control intervention, with distribution prioritized by endemicity. Nevertheless, owing to insecticide resistance, the risk of malaria remains a challenge in most parts of the country [10]. Approximately 3,294,000 malaria cases and more than 11,000 malaria-related deaths were reported in Kenya in 2023 [4]. Malaria transmission is primarily mediated by the *Anopheles gambiae* species complex and the *Anopheles funestus* group of mosquito vectors. Among the *An. gambiae* species complex, *An. arabiensis* is predominant in Kenya because of its ability to thrive in arid and semiarid regions [11].

*An. arabiensis* is phenotypically and genetically diverse, suggesting its long-standing presence and potential for adaptation to environmental changes such as the introduction of insecticides [12]. This malaria vector has also shown a recent resurgence in Kenya compared with *An. gambiae* [13]. *An. arabiensis* exhibits exophilic (resting outdoors) and exophagic (feeding outdoors) behavior, increasing its indoor and outdoor transmission potential by evading most vector control interventions that are primarily deployed indoors [14, 15]. Its difference in ecology may explain why comparatively less insecticide resistance has been observed in *An. arabiensis* compared to indoor resting *An. gambiae* and *An. coluzzii* [16, 17] Whereas *An. arabiensis* is typically anthropophilic, its counterbalanced zoophilic behaviour can also enhance survival by allowing access to alternative blood meal sources [15]. Resting outdoors could expose the malaria vector to insecticides deployed for pest control in livestock and poultry farming systems, increasing the risk of exposure and thus developing cross-resistance mechanisms [18]. The impact of agricultural insecticides on such mechanisms is little understood in Kenya but has been reported from Northern Cameroon where metabolic resistance increased in *An. arabiensis* after pyrethroid insecticide application in an area of extensive cotton cultivation [19].

While insecticide resistance in the *An. gambiae* complex has been extensively researched, there is comparatively limited information on the resistance profiles of *An. arabiensis* in Kenya [12, 20, 21]. In general, insecticide resistance in mosquitoes is driven by target site resistance and metabolic resistance mechanisms. Target site resistance involves mutation of a gene encoding a protein that is the binding target of an insecticide, such as the voltage-gated sodium channel, acetylcholinesterase (AChE), and γ-aminobutyric acid (GABA) receptors, which can confer resistance to various classes of insecticides. For example, the *Vgsc*-L995F/L995S mutation in the voltage-gated sodium channel is associated with resistance to dichloro-diphenyl-trichloroethane (DDT) and pyrethroids in *An. gambiae* s.s [20]. and *An. coluzzii* [21] in Kenya. Alterations of *Ace-1*-G280S in AChE and the *rdl*-A296S/A296G mutation in GABA receptors have been linked to organophosphate, carbamate, cyclodiene and fipronil resistance in *An. gambiae* s.s. and *An. coluzzii* reported in several regions of Sub-Saharan Africa [22–25]. In contrast, metabolic resistance involves a variety of detoxification mechanisms, such as increased expression or efficacy of the gene that metabolizes insecticides [26]. Copy number variations (CNVs) involve gene duplication and/or deletion of genes encoding these enzymes, such as cytochrome P450, carboxylesterase, and glutathione S-transferase, which increase their expression levels, leading to increased insecticide metabolism and resistance in *An. gambiae* s.l [27, 28].

This study investigated the genomic landscape of insecticide resistance in *An. arabiensis* populations from four malaria endemicity regions in Kenya via whole-genome sequencing data. Specifically, this study characterized the population structure of *An. arabiensis* across Kenya, identified and quantified known and novel insecticide resistance mechanisms and examined the geographical distribution of these resistance mechanisms.

## Methods

This study employed whole-genome sequencing and bioinformatic analyses to investigate the population structure and insecticide resistance mechanisms of *An. arabiensis* in Kenya.

### Mosquito sampling

Mosquitoes were collected from four malaria endemicity regions in Kenya. These included two western locations close to the border with Uganda, namely, Kakuma in the arid northern county of Turkana characterized as seasonal malaria epidemic region and Teso District which refers to the current Teso South and Teso North sub counties in Busia County near Lake Victoria classified as the lake malaria endemic region. Locations in the Highlands of central Kenya were Mwea in Kirinyaga County and Thika in Kiambu County categorized as low-risk malaria region. A further collection was made on the southeast coast of Kenya in Kwale County classified as the coast malaria endemic region [10]. The distance between the four malaria endemicity regions selected for this study is at least 500 km apart. In addition, whole-genome sequence data generated during phase 3 of the Ag1000G project [29] was available for Kilifi County, which is located on the north coast in the coast malaria endemic region. The sampling scheme encompassed contrasting ecological zones since central Kenya experiences a cool and wet climate that favours agricultural activity, whereas Turkana in the northwestern part of the country has an arid and semiarid climate with nomadic pastoralism. Teso, Kilifi and Kwale experience hot and wet climates with subsistence agricultural activities [9].

All study sites have historically received standard pyrethroid-treated mosquito nets distributed through mass campaigns conducted every three years until 2021 [10]. In response to emerging pyrethroid resistance in local vector populations, Busia County where Teso is located implemented a strategic shift to piperonyl butoxide (PBO) nets in 2021, followed by the introduction of annual IRS using pirimiphos-methyl [30]. These vector control interventions informed our selection of insecticides for testing: deltamethrin and alpha-cypermethrin (pyrethroids used in ITNs) and pirimiphos-methyl (an organophosphate used in IRS throughout Kenya). The Kakuma refugee camp in Turkana County presents a particularly significant case, with its exceptionally high population density of 12,000–13,000 people per square kilometer, potentially creating conditions for accelerated insecticide resistance development due to intensified insecticide exposure [31].

Mosquito larvae were collected from across multiple oviposition sites using standard dippers. The samples were transported to Kenya Medical Research Institute insectary in containers labelled with the site and date of collection. The larvae were maintained on fine ground powder of sea Vipan staple diet™ (Sera Germany) fish food and reared at a temperature of 27 ± 2 °C and humidity of 80 ± 10%. Pupae were transferred to a cup with water inserted in labelled mosquito cages and maintained on a 10% sugar solution. Bioassay experiments were carried out for the most recent collections made between 2019 and 2021 to best reflect contemporary insecticide resistance. A CDC bottle bioassay was conducted on 3 to 5-day-old adult female mosquitos, as described by Brogdon and Chan [32]. In brief, mosquitoes were evaluated for resistance after 30 min of exposure to a diagnostic dose of 12.5 µg/ml deltamethrin, 12.5 µg/ml alpha-cypermethrin and 20 µg/ml pirimiphos methyl and later transferred to paper cups with cotton wool soaked in 10% sugar solution and observed for up to 72 h. Adult mosquitoes were identified as members of the *An. gambiae* complex via a morphological key [33] before being stored in ethanol for whole-genome sequencing.

### Sequencing and variant calling

The sequencing and SNP protocols were followed as previously described [21] and followed the guidelines provided by the Ag1000G project [29]. Whole-genome sequencing was performed via an Illumina HiSeq 2000/X, which generated 100–150 bp paired-end reads. Alignment and SNP calling were performed in reference to the AgamP4 genome using BWA v0.7.15 and GATK v3.7.0. Only high-quality data from individual vector samples with > 10X median coverage, a large proportion of non-missing data (> 80% across the genome), a low contamination threshold (< 4.5%) and passing site filters that excluded sites with less reliable SNP calling and genotyping were retained for analysis.

Haplotype phasing was performed using both read-backed phasing using WhatsHap v1.0 and statistical phasing via SHAPEIT v4.2.1using a pipeline available in WDL implementation [34].

CNV calling followed the methods described in [26]. Briefly, the copy number state was inferred across 300 base pair windows of the genome for each *An. arabiensis* individual samples on the basis of their normalized coverage data using a Gaussian hidden Markov model (HMM) implemented in hmmlearn (https://github.com/hmmlearn/hmmlearn). A CNV call was identified as a contiguous run of at least five genomic windows with a predicted copy number > 2 or > 1 for chromosome X in males. CNV calls were filtered such that only those with a high likelihood > 1000, as predicted by the HMM, were retained. Individuals with high coverage variance greater than 0.35 were removed to increase reliability.

### Species identification

The samples analyzed in the present study were previously identified to species via a set of Ancestry Informative Markers (AIMs) as previously described [21]. The AIMs are a set of 700 SNPs described by the Ag1000G project and are able to distinguish between *An. arabiensis*, *An. gambiae* and *An. coluzzii*. These individuals were further confirmed as *An. arabiensis* with both Principal component analysis (PCA) and neighbor-joining trees [21] because they are grouped separately from other members of the *An. gambiae* complex.

### Geographical population structure

To investigate the population structure of *An. arabiensis* across Kenya, 100,000 biallelic SNPs with a minor allele count > 0.2 and no missing data were used. These SNPs were equally distributed across a region of chromosome three unaffected by structural variants such as inversions. A PCA was performed on the allele counts of these SNPs for the samples collected in this study and data that was available as part of the Ag1000G project. Summary statistics of nucleotide diversity (π), Watterson’s Theta (θ), Tajima’s *D* and Hudson’s pairwise FST for population cohorts with at least ten individuals were calculated via built-in functions within the malariagen_data python package [35].

### Insecticide resistance

To investigate the genomic variation, present at known insecticide resistance loci, the analysis focused on the mosquito cohorts tested in the bioassay experiment. The frequencies of amino acid substitutions were calculated separately for individuals who survived or died after insecticide treatment. Frequencies were calculated based on observations of nonsynonymous SNPs given transcripts for the voltage-gated sodium channel (Vgsc; AGAP004707), the glutathione S-transferase gene conferring resistance to DDT (*Gste2*; AGAP009194), the resistance to dieldrin gene (*Rdl*; AGAP006028) and the organophosphate target gene acetylcholinesterase (*Ace1*; AGAP001356). Only amino acid changes greater than 5% frequency were reported, as resistance alleles under positive selection are unlikely to be at a lower frequency. We also generated the frequencies of copy number variants (CNVs) for genes associated with insecticide resistance for the same cohorts. These genes included the cytochrome P450 genes (AGAP002862–AGAP002870, AGAP000818, AGAP008212–AGAP008219), carboxylesterases (AGAP006228, AGAP006723–AGAP006728), *Ace1* (AGAP001356) and *Gste2* (AGAP009194). Only CNVs with a frequency greater than 5% were retained.

The associations of the observed variants with the bioassay outcome, i.e., dead or alive for each bioassay experiment tested separately for each population cohort, were formally tested via the Fisher exact test. Population cohorts for each bioassay outcome from Kwale were excluded from this analysis because it had fewer (< 5) samples. The test was either based on SNP allele counts at a specific locus or on the distribution of CNV counts across a gene region. Analyses were performed using the malariagen_data and SciPy python packages.

### Selection scans

To investigate selection signals, H12 homozygosity statistic was calculated as defined by [36] across windows of the genome using the malariagen_data python package. The optimal window size was chosen for each population cohort by plotting the values produced for each window size and identified the distribution of H12 values below 0.1 for the 95th percentile. The statistical peaks in H12 represent either a hard or soft sweep. We also plotted the difference in the observed H12 values between individuals who either survived or died in each bioassay experiment. A significant peak in H12 difference indicates differential selection acting on the cohorts.

### Diplotype clustering

Hierarchical clustering was performed on either haplotypes or diplotypes using city block genetic distance and complete linkage. Diplotype clusters with low heterozygosity are suggestive of a haplotype under selection. To investigate variants associated with diplotypes, the observed amino acid substitutions and CNV variants were plotted onto the resulting dendrogram.

## Results

### Population sampling

**Fig. 1:**
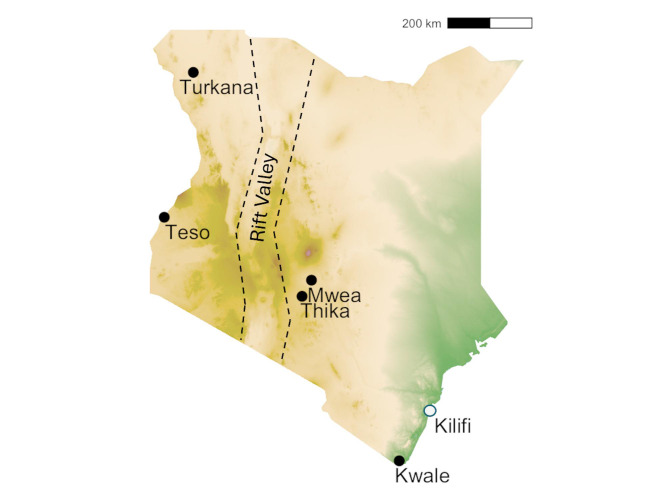
Map of Kenya showing the country’s elevation and sample collection locations developed via geospatial country data from the geographic information system of the commission (GISCO) using the R package giscoR

A total of 498 samples of *An. arabiensis* mosquitoes were collected from five locations in Kenya between 2007 and 2021 (Fig. 1, Supplementary Table 1). Insecticide treatment and bioassay results are summarized in Table 1, with detailed methods described in [21]. The samples were previously identified as *An. arabiensis* based on AIMs, and their taxonomic status was confirmed with both PCA and neighbor-joining trees with other known taxa. These mosquitoes had a median genome coverage between 11 and 80X, resulting in 156,787,860 SNPs aligned to the reference passing site quality filters as defined by the Ag1000G phase 3 project. A total of 27,241,041 biallelic SNPs segregated within the sample set.

**Table 1:** Sampling locations and the number of mosquitoes successfully sequenced for each. Mosquitoes tested within a bioassay experiment before sequencing were recorded as alive or dead based on the treatment outcome

				Insecticide treatment and Bioassay Outcome						
				alpha-cypermethrin		Deltamethrin		pirimiphos methyl		Untested
Location	Year	Latitude	Longitude	Alive	Dead	Alive	Dead	Alive	Dead	
Kwale	2019	−4.572	39.257	0	0	2	3	1	1	0
Mwea	2007	−0.717	37.382	0	0	0	0	0	0	18
	2014	−0.717	37.382	0	0	0	0	0	0	15
	2020	−0.717	37.382	18	28	0	0	0	26	0
	2021	−0.717	37.382	0	0	16	13	16	0	0
Teso	2013	0.626	34.236	0	0	0	0	0	0	23
	2019	0.626	34.236	4	19	4	13	6	10	0
Thika	2019	−1.061	37.181	0	0	22	15	11	8	0
Turkana	2006	3.717	34.857	0	0	0	0	0	0	19
	2019	3.717	34.857	17	34	29	24	37	33	0

### Geographical population structure

**Fig. 2:**
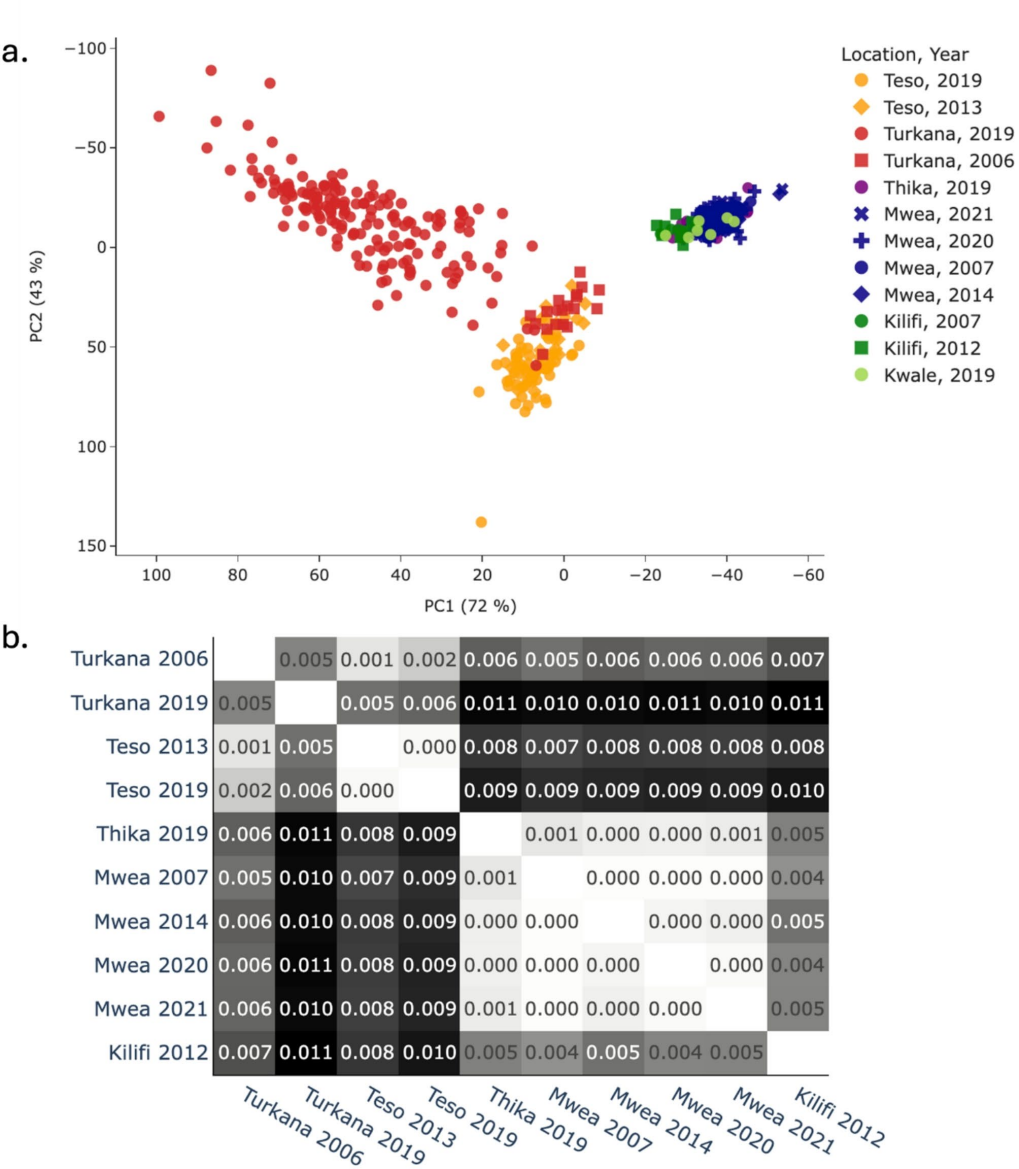
Investigation of the population structure of Kenyan *An. arabiensis* using (**a**) PCA and (**b**) Measures of genetic differentiation (FST)

To explore the geographical population structure among *An. arabiensis* from Kenya, data from the present study was combined with 13 individuals from Kilifi on the northern coast of Kenya, previously available in the Ag1000G phase 3 resource. A PCA was performed using 100,000 biallelic SNPs on a region free from polymorphic inversions on chromosome three (3 L:15,000,000–41,000,000). The PCA revealed two major groupings (Fig. 2a). One group consisted of mosquitoes from Mwea and Thika from the central locations and Kilifi and Kwale on the northern and southern coasts, respectively. Another group was composed of the northwestern populations of Turkana and Teso, although individuals collected from Turkana in 2019 formed a somewhat separate and dispersed group close to the cluster. Further investigation via the generation of genetic diversity summary statistics revealed that *An. arabiensis* population cohorts from northwestern Kenya presented higher values of genetic diversity and lower Tajima’s D values than those from central eastern Kenya (Supplementary Fig. 1). This was particularly true of the 2019 collection from Turkana, which formed a separate grouping on the PCA, suggesting that the region may be subject to population expansion. Population structure was explored by calculating FST between population cohorts with a minimum of ten individuals, and a pattern concurrent with the findings from the PCA was found. There was a greater FST between northwest, i.e., Turkana and Teso, and central-coastal locations, i.e., Mwea and Kilifi (FST 0.005–0.011, Fig. 2b), than within these regions (FST 0.000–0.005, Fig. 2b). Overall, this study suggests restricted gene flow between the northwestern region and the rest of Kenya.

To explore whether the regional structure observed in Kenya extends to surrounding countries, a PCA including *An. arabiensis* from neighbouring Uganda and Tanzania, which are publicly available in the Ag1000G phase 3 repository, were used. Three major groupings were apparent (Supplementary Fig. 2). One group included samples from northwestern Kenya and Uganda on the western side of the Rift Valley, whereas the other included samples from the central and coastal regions of Kenya and Moshi in eastern Tanzania. A third intermediate group included samples from Muleba and Tarime in northwestern Tanzania, which are located on the western side of the Rift Valley and south of Lake Victoria. Therefore, restricted gene flow in *An. arabiensis* extends to populations separated by major geographical barriers. In support of this notion, genomic differentiation was greater between central eastern Kenya and Uganda (FST 0.009–0.010, Supplementary Fig. 3) than between northwestern Kenya and Uganda (FST 0.000–0.006, Supplementary Fig. 3). Furthermore, the greatest degree of genomic differentiation between Kenya and Tanzania was observed between Turkana in Kenya and Moshi in central eastern Tanzania (FST 0.004–0.009, Supplementary Fig. 3) rather than between Muleba and Tarime in northwestern Tanzania (FST 0.000–0.006, Supplementary Fig. 3).

### Insecticide resistance

#### Target site resistance

**Fig. 3:**
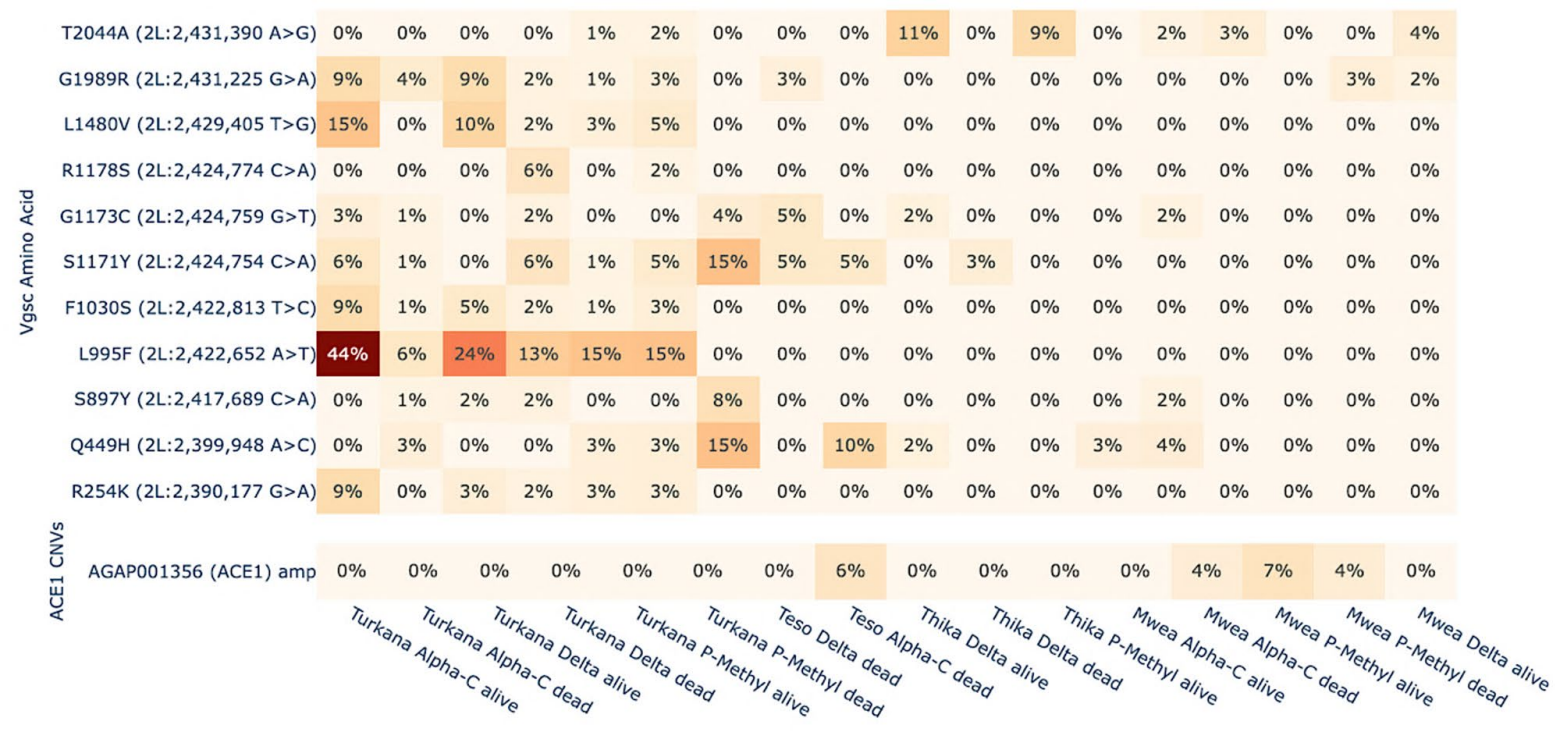
Investigation of the molecular mechanisms of target-site insecticide resistance, including amino acid frequencies at the voltage-gated sodium channel and CNV frequencies at ACE-1

To determine the geographical distribution of target site resistance across Kenya, the frequencies of amino acid mutations at major insecticide binding regions, including the voltage-gated sodium channel *Vgsc* (AGAP004707), resistance to dieldrin *Rdl* (AGAP006028) and acetylcholinesterase *Ace1* (AGAP001356), were calculated. The data analyzed were population cohorts of *An. arabiensis* tested within the replicate bioassay experiments using either alpha-cypermethrin, deltamethrin or pirimiphos methyl. Amino acid frequencies were calculated separately for the cohorts that either experienced mortality or survived each bioassay treatment for comparison. Among the target site mutations that confer insecticide resistance, only the Vgsc-L995F substitution was observed at appreciable frequencies in Turkana, northwestern Kenya (13–44%) (Fig. 3). The L995F mutation has been associated with resistance to DDT and pyrethroids [20]. To investigate whether the L995F mutation could explain the observed resistance to pyrethroids in Turkana, differences in SNP allele counts were tested for significant association with bioassay outcome using Fisher exact test. The frequency of L955F was significantly higher in alpha-cypermethrin alive mosquitoes from Turkana versus those that died (*P* = 9.12e-6), (Supplementary Table 2). Other comparisons were non-significant.

The presence of L995F in Kenya was interesting given that it is generally associated with West and Central African populations of *An. gambiae* and *An. coluzzii*. Therefore, the presence of L995F in other *An. arabiensis* populations in the Ag1000G phase 3 dataset was explored. The L995F mutation was not detected in other East African populations, including Uganda, Tanzania and Malawi, but was detected in Burkina Faso, West Africa (Supplementary Fig. 4). However, owing to the low number of samples of only three *An. arabiensis* from Burkina Faso, it was not possible to determine whether the haplotype background of L995F in East Africa was similar and therefore originated from West African *An. arabiensis*. Recently, the presence of L995F in *An. coluzzii* was reported from the same region of Turkana, Kenya, where the mutation in *An. arabiensis* was found [21]. It is therefore possible that there has been adaptive introgression between the two species. Evidence for adaptive introgression was assessed using haplotype clustering of the Vgsc gene for *An. arabiensis* and *An. coluzzii* from the Turkana region. The two species formed separate haplotype clusters, indicating that no haplotype sharing occurred between the species and ruling out the possibility of adaptive introgression (Supplementary Fig. 5).

The copy number variants (CNVs) at the Ace1 locus, which is the target site of organophosphate and carbamate insecticides, have not yet been identified in *An. arabiensis*. However, the current study found Ace1 CNVs were present at low frequencies in samples from Mwea (Fig. 3). A duplication at this locus is usually associated with the G280S (G119S) SNP because it restores fitness in the absence of insecticides by allowing retention of the wild type [24]. However, the G280S SNP was not observed, nor was there evidence for selection at the locus based on selection scans with the H12 statistic (Supplementary Fig. 6, see Results subsection). Therefore, it is possible that a CNV in this region is either transient and/or subject to a fitness trade-off that restricts duplication.

#### Metabolic resistance

To investigate the geographical distribution of metabolic resistance, the frequencies of copy number variants at loci previously associated with insecticide resistance, including Cytochrome P450s (AGAP002862-AGAP002870, AGAP000818, AGAP008212-AGAP008219), carboxylesterases (AGAP006228, AGAP006723-AGAP006728) and glutathione S-transferase *Gste2* (AGAP009194), were calculated.

#### Cytochrome P450s

**Fig. 4:**
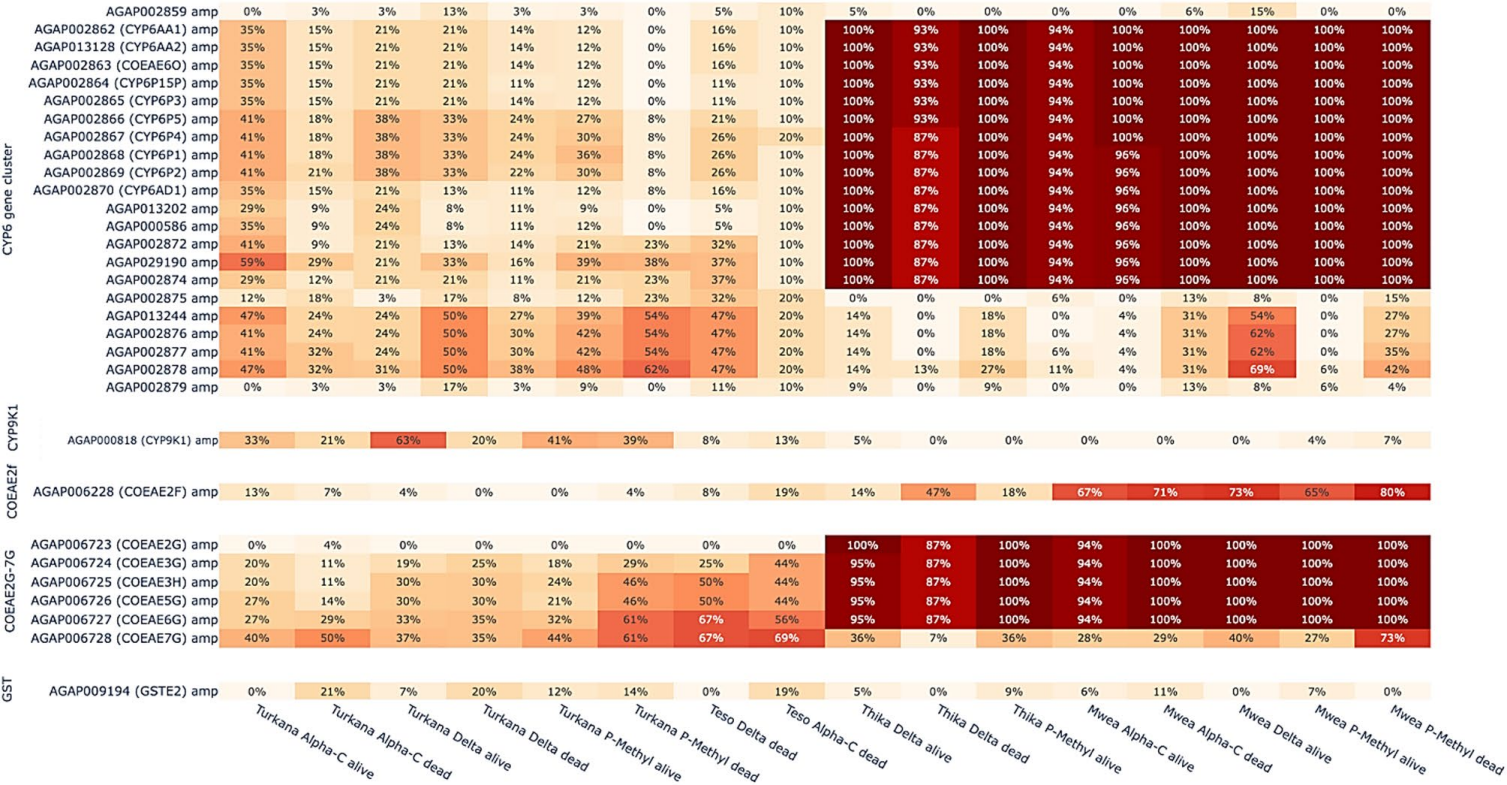
Investigation of the molecular mechanisms of metabolic insecticide resistance, including CNVs at the cytochrome P450 gene cluster Cyp6aa/p and Cyp9k1 and the carboxylesterases Coeae2f and Coeae2g-7 g and Gste2

**Fig. 5:**
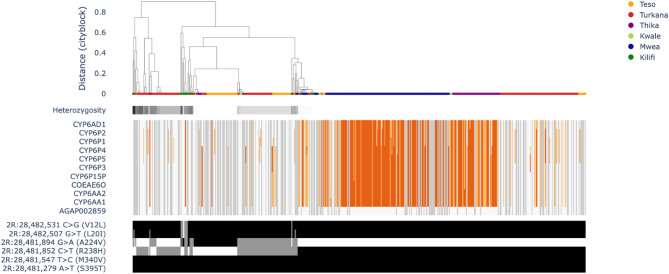
Diplotype clustering of the Cy6aa/p gene cluster. The clustering dendrogram is accompanied by heterozygosity, with darker shades representing higher diversity and copy number variant frequencies, with darker shades of red indicating a higher copy number. Clusters of identical diplotypes with low heterozygosity are expected to be under selection. The substitutions present in each individual are shown below with blocks of the same colour indicating a shared variant

Copy number amplifications at the Cyp6aa/p cytochrome P450 gene cluster were present at all locations. These included CNV amplifications at Cyp6aa1 and Cyp6aa3, which were previously implicated in cross-resistance to pyrethroids and carbamates in *Anopheles* mosquitoes [1]. Copy number variant amplification frequencies at these loci were particularly high in the central region of Kenya, reaching fixation in Mwea and Thika (Fig. 4). The distribution of CNV amplification across the Cyp6aa/p gene cluster was tested for differences based on the bioassay results via the Fisher exact test. The results had a significant association between copy number at the CYP6 genes and both alpha-cypermethrin and deltamethrin survival in Turkana (*P* = 6.05e-98, *p* = 5.76e-57, respectively) and deltamethrin survival in Thika (*P* = 3.42–49) (Supplementary Table 2). In addition, there was a significant association with pirimiphos methyl in Turkana, Teso and Thika (*P* = 0.38e-07, *P* = 1.13e-05, *P* = 6.74e-07), indicating a possible role in cross-resistance across Kenya. Recently, a SNP at Cyp6p4 (I236M) in the Cyp6aa/p cluster was associated with high resistance to pyrethroids in tandem with a CNV duplication at Cyp6aa1 and a transposable element upstream of this variant [37]. Therefore, diplotype clustering of the locus was used to investigate whether any unique SNPs were observed in the variants under selection. Diplotype clustering of the locus was also used to determine whether northwestern and central Kenya were subject to the same resistance mechanism. The diplotype clustering revealed two major groups with low heterozygosity and high frequency in the population, indicating that these variants were under selection (Fig. 5). Marked differences were apparent between the two diplotype clusters. Copy number variants were generally not observed for the first diplotype, and a SNP unique to the cluster was present at A224V. In contrast, the second cluster was commonly amplified and did not possess any unique SNPs. Therefore, multiple insecticide resistance mechanisms at the Cyp6aa/p cluster involving both nucleotide mutations and CNV amplification potentially increase resistance in Kenya. Both diplotypes were found in individuals from across Kenya, suggesting that resistance mechanisms are shared among geographic locations.

Cyp9k1 has also been implicated in the detoxification of pyrethroids such as deltamethrin and DDT [38]. Copy number variant amplifications at Cyp9k1 were present across Kenya; however, in contrast to our findings for the Cyp6aa/p cluster, these amplifications occurred at a low frequency in central Kenya (< 7%), although significantly associated with deltamethrin and pirimiphos methyl in Thika (Fig. 4, Supplementary Table 2). High frequencies of up to 63% were observed in Turkana. A significant association of Cyp9k1 CNVs with mosquitoes from Turkana that survived the deltamethrin and alpha-cypermethrin bioassay (*P* = 5.08e-06, *P* = 0.01, respectively) was also observed, suggesting that Cyp9k1 CNVs could increase pyrethroid resistance in the region.

Another cytochrome P450 that enhances pyrethroid resistance in *An. gambiae* and therefore potentially *An. arabiensis* was CYP6Z. This locus is also known to metabolize carbamates and insect growth regulators [39]. However, only low CNV frequencies of CYP6Z2 across Kenya were observed (Supplementary Fig. 7). Overall, cytochrome P450 at the Cyp6aa/p gene cluster and CYP9K1 are metabolic resistance mechanisms of interest in *An. arabiensis* from Kenya.

#### Carboxylesterases

Copy number variant amplification at the carboxylesterase Coeae2f and Coeae2g-7 g gene cluster have been associated with resistance to the organophosphate pirimiphos-methyl [27, 28]. High frequencies of CNV amplification at both loci in the central regions of Mwea and Thika were observed, with frequencies reaching 80% for Coeae2f and 100% for genes in the Coeae2g-7 g cluster, respectively (Fig. 4). While most variants at the Coeae2g-7 g cluster were fixed or close to fixation for all central Kenyan cohorts, the frequencies of Coeae2g-7 g differed in northwestern Kenya. Amplifications at this locus were significantly associated with deltamethrin and pirimiphos methyl survival in central Kenya (Supplementary Table 2) Additionally, amplifications at Coeae2f were associated with alpha-cypermethrin survival in Turkana (*P* = 4.50e-09).

#### Glutathione S-transferase

Duplications at the glutathione S-transferase (Gste2) target site have been associated with organophosphate resistance and SNP variants, with a fitness cost in *An. gambiae* and *An. coluzzii* [40]. The CNVs at the locus were found across Kenya at appreciable frequencies of up to 21%, but the associated SNP (I114T) was not observed, nor was there a signal of selection based on the H12 statistic (Supplementary Fig. 8, Fig. 4, see Results subsection Selection). Overall, genomic evidence suggests that organophosphate resistance in Kenya is largely driven by carboxylesterases rather than the overexpression of GSTE2 or variation at the target site ACE1.

#### Candidate loci under selection

To investigate the evidence for novel candidate genes conferring insecticide resistance, the presence of loci under selection were sought. To do so, the H12 homozygosity statistic across windows of the genome was calculated, and peaks in H12 values were identified. The analysis was performed on chromosomes 2 and 3 only since the X chromosome is particularly divergent from the *An. gambiae* reference genome. As expected, a signal of selection was observed at known resistance loci where variants of interest were also observed (Supplementary Figs. 9 to 12). These included the Vgsc gene for Turkana cohorts where the L995F substitution was observed, although small peaks were also present for populations from Thika and Mwea, where this variant was not observed. In addition, a selection signal was observed in all population cohorts at the Cyp6aa/p gene cluster, which is associated with widespread CNVs. A signal of selection was also observed at both Coea2f and the Coeae2g-7 g gene cluster in all cohorts from central Kenya Mwea and Thika only, where high CNV frequencies were observed.

**Fig. 6:**
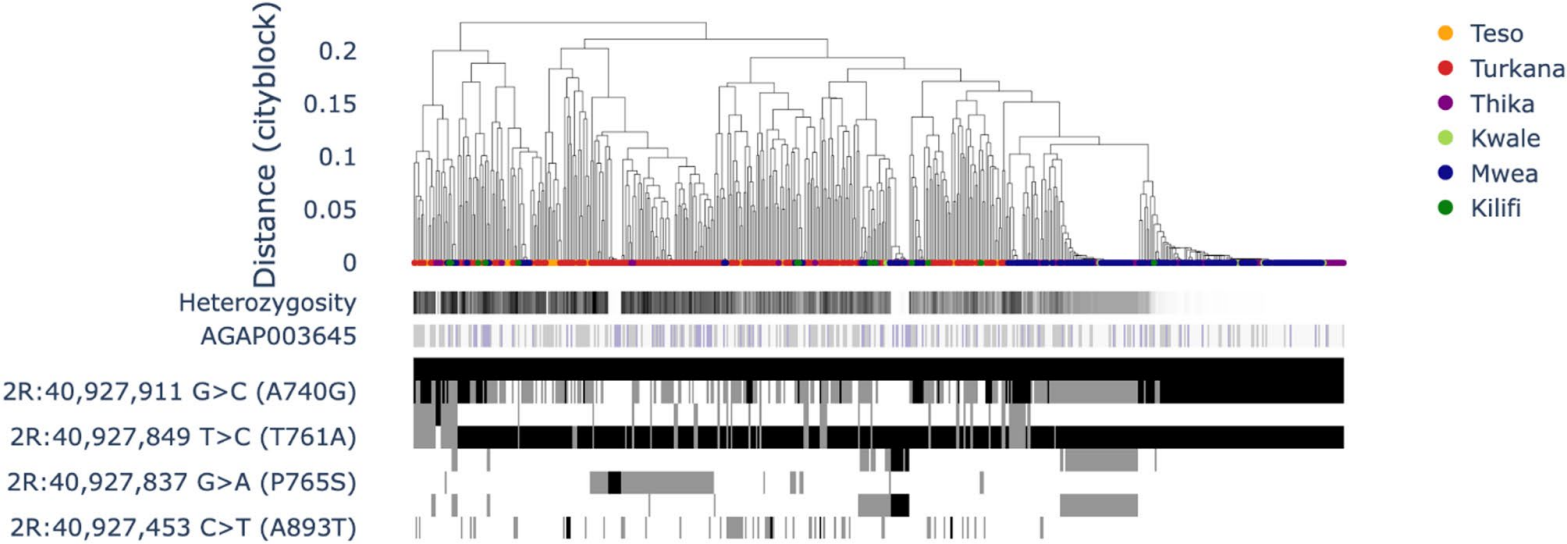
Diplotype clustering of the Keap1 gene. The clustering dendrogram is accompanied by heterozygosity for the region, with darker shades representing higher diversity and copy number variant frequencies, with darker shades of purple indicating a higher copy number. Clusters of identical diplotypes with low heterozygosity are expected to be under selection. The substitutions present in each individual are shown below with blocks of the same colour indicating a shared variant

In addition to known loci, a selection peak was observed at the Keap1 gene (AGAP003645: 2R:40,926,195 − 40,945,169) for central cohorts from Mwea and Thika but not for cohorts originating from the northwestern and coastal regions (Supplementary Figs. 8 and 9). The importance of the Keap1 gene for insecticide resistance is noteworthy because it regulates the transcription factor Maf-S, which is known to trigger the expression of multiple metabolic resistance genes, including cytochrome p450s and glutathione S-transferases, in response to oxidative stress [41]. Since a particular variant has not yet been associated with Keap1, further investigation using diplotype clustering was performed to assess whether either a CNV duplication or SNP was associated with the candidate diplotypes under selection. Three diplotypes with low homozygosity (Fig. 6) were observed. Among the three diplotypes, only one was unique to central Kenya, where a strong signal of selection was observed. Interestingly, this diplotype had a unique set of SNPs, including E762 and D780N. One substitution (E762*) was a stop gain mutation, indicating a potential loss of function. The role of Keap1 within its transcriptional pathway is to act as a repressor that prevents the unnecessary expression of detox genes in the absence of stress [41]. Therefore, it is plausible that a loss of function could result in elevated antioxidant defence. These variants are candidates under selection that could form the focus of future validation studies.

## Discussion

*Anopheles arabiensis* is the predominant malaria-transmitting vector in Kenya [11], yet our understanding of the underlying population structure of the malaria vector and insecticide resistance mechanisms remains limited. This study analyzed the whole-genome sequence data of 498 *An. arabiensis* individuals across five regions in Kenya, revealing substantial heterogeneity in population structure and insecticide resistance profiles.

Our analysis revealed a distinct geographical population structure of *An. arabiensis* in Kenya. The principal component analysis (PCA) and F_ST_ revealed that samples from central Kenya (Mwea and Thika) were genetically similar to those from coastal Kenya (Kilifi and Kwale) but differentiated from those collected in the northwest (Turkana and Teso). The observed population structure aligns with previous studies highlighting the Great Rift Valley as a significant factor in genetic differentiation between populations from western and coastal Kenya of *An. gambiae* [12] and *An. funestus* [42]. Additionally, our findings suggest that Lake Victoria impedes gene flow, which is consistent with previous research on *An. gambiae* populations from islands in the lake [43, 44].

Variation in natural ecological factors, including climate and land use, such as human settlement patterns and agricultural activity across the country, are alternative or additional factors that may influence mosquito population structure and gene flow. The heterogeneity in climate suggests that populations across the country may be unequally connected by habitat suitability. The Turkana population in particular exhibited higher genetic diversity and lower Tajima’s D values, suggesting a different demographic history, including population expansion and a larger effective population size. This could reflect the arid environment of the region, to which *An. arabiensis* is well adapted [11], or potentially that the population is less impacted by vector control efforts. Future research should focus on investigating the specific ecological factors driving population structure and genetic diversity and their implications for malaria transmission.

The results of the present study revealed distinct insecticide resistance patterns across the regions potentially influenced by restricted gene flow. For example, genome-wide selection scan analysis and CNV amplification of the Cyp6aa/p cytochrome gene family indicated a strong selection signal and high CNV frequencies in central Kenya compared with northwestern Kenya. Previous studies have reported an association of high CNV frequencies in Cyp6aa/p cluster genes with metabolic resistance in *An. gambiae* from Kenya [37], *An. coluzzii* from southern Cote d’Ivoire [45] and *An. arabiensis* from Tanzania [27]. Additionally, the association of CNVs we observed with pirimiphos methyl, alpha-cypermethrin and deltamethrin suggests a potential role in cross resistance across Kenya. In particular, CNV amplifications at Cypaa1 and Cyp6p3, which were previously implicated in cross-resistance to pyrethroids and carbamates in *An. gambiae* mosquitoes, were fixed in the central region of Kenya [1]. Despite the frequency differences observed, the two major Cyp6aa/p haplotypes apparently under selection in *An. arabiensis* were shared across regions, implying that they should elicit a similar molecular response. However, further sampling may determine fine-scale heterogeneity in the different mechanisms and increase statistical power to test for an association with the bioassay outcomes. Additionally, higher CNV frequencies in the carboxylesterase Coeae2f and Coeae2g-7 g gene clusters were observed in the central region than in northwest Kenya. This gene amplification has been previously associated with resistance to the organophosphate pirimiphos-methyl [27, 28]. Furthermore, the results of the present study indicated that CNV amplification of the Coeae2g-7 g gene in central Kenya was significantly associated with deltamethrin and pirimiphos methyl survival. The high organophosphate resistance observed in a region that receiving pyrethroid only nets for malaria vector control [46], is likely attributed to intensive agricultural pesticide use in the region, particularly in areas with large-scale rice cultivation (Mwea) and horticultural farming (Thika) [11]. Despite the limited research in agricultural pesticide application in central Kenya, studies have shown that tomato farmers in Mwea rely heavily on multiple insecticide classes including pyrethroid, carbamate organophosphates and neonicotinoids for pesticide management. Furthermore, these investigations revealed that farmers frequently deviate from recommended pesticide application protocols in Mwea, potentially exacerbating selection pressure for resistance development in local mosquito populations [47, 48].

In contrast to the central region, northwestern Kenya, particularly Turkana, presented appreciable frequencies of the Vgsc-L995F mutation and Cyp9k1 duplications, which are associated with pyrethroid and DDT resistance [16, 38]. In particular, the significant association of Cyp9k1 CNVs with survival in the deltamethrin and alpha-cypermethrin bioassays suggested that *An. arabiensis* in the Turkana region could have increased resistance to pyrethroids. This resistance profile likely emerges from intense selection pressure in the region, where high population density has necessitated widespread deployment of pyrethroid-treated nets. These conditions have potentially created a resistance hotspot [31]. As a result, alternative insecticides or integrated vector control method may be necessary to address emerging resistance in this region. The presence of the L995F mutation in Turkana is noteworthy, as it is generally associated with West and Central African populations of *An. gambiae* and *An. coluzzii* [16], and we did not observe the allele in other East African *An. arabiensis*. Our analysis ruled out the adaptive introgression of Vgsc mutations between *An. arabiensis* and *An. coluzzii* in Turkana, suggesting independent evolution of resistance mechanisms and highlighting a further need to investigate its origin.

Genome-wide selection scans have been instrumental in identifying novel genes under divergent selection in malaria vectors. The current analysis of *An. arabiensis* in Kenya revealed a signal of positive selection in the Keap1 gene at the 2R chromosome from the central Kenya region. Previous research on *Drosophila melanogaster* has shown that the Keap1 gene is overexpressed in the presence of deltamethrin, whereas cytochrome P450 family genes are significantly downregulated, highlighting the important role of the Keap1 gene in the detoxification of pyrethroids [49]. Keap1 is part of a regulatory pathway involving the *Maf-S* and *cnc* genes, which triggers the upregulation of metabolic enzymes in response to oxidative stress [41].

The results of the present study revealed three distinct diplotypes in the Keap1 gene region with low heterogeneity, suggesting that these diplotypes were under selection. One diplotype cluster unique to central Kenya carried a stop gain mutation (E762*). This mutation could lead to loss of function, resulting in elevated antioxidant defense and increased basal expression of these detoxification enzymes. These findings suggest that Keap1 may play an important role in mediating insecticide resistance. These variants identified in the Keap1 gene are promising candidates for future validation studies to elucidate their specific role in insecticide resistance. Functional studies, such as those conducted in *Drosophila melanogaster*, can help evaluate the effects of these mutations on insecticide resistance [45].

## Conclusion

The observed high heterogeneity in population genetic structure and diverse insecticide resistance mechanisms across Kenya is likely driven by restricted gene flow in tandem with ecological and land use variables across the country. This complexity necessitates region-specific tailoring of malaria vector control strategies rather than a one-size-fits-all approach. The emergence of novel variants, coupled with the high frequencies of known resistance variants, raises concerns about the long-term efficacy of current malaria vector control interventions and the potential pressure introduced by new vector control tools.

This study underscores the value of integrating genomics surveillance into routine national malaria control programs. Specifically, routine whole genome sequencing of mosquito samples collected during standard entomological surveillance and phenotypically tested for insecticide resistance would enable continuous monitoring of emerging resistance variants. Additionally, incorporating genomic analysis into trials of new interventions can help identify where insecticide resistance might compromise effectiveness. National programs should build capacity in vector genomics and bioinformatics to monitor resistance patterns and guide evidence-based selection of appropriate control interventions.

## Supplementary Information

Supplementary Material 1: Supplementary Figure 1. Bar plots of genetic diversity statistics for population cohorts, including nucleotide diversity (π), Watterson’s Theta (θ)and Tajima’s *D*.

Supplementary Material 2: Supplementary Figure 2. PCA to investigate the population structure of *An. arabiensis* from Kenya in relation to the neighbouring countries of Uganda and Tanzania.

Supplementary Material 3: Supplementary Figure 3. Investigation of the genetic differentiation of population cohorts with Hudson’s pairwise FST

Supplementary Material 4: Supplementary Figure 4. Investigation of the frequencies of the voltage-gated sodium channel substitution L995F in African *An. coluzzii*.

Supplememtary Material 5: Supplementary Figure 5. Investigation of haplotype sharing with hierarchical clustering of the voltage-gated sodium channel gene

Supplementary Material 6: Supplementary Figure 6. Investigation of amino acid substitutions at the ACE-1 locus

Supplementary Material 7: Supplementary Figure 7. Investigation of CNV frequencies in the Cyp6z/m gene cluster

Supplementary Material 8: Supplementary Figure 8. Investigation of amino acid substitutions at the GST locus

Supplementary Material 9: Supplementary Figure 9. Investigation of signals of selection with the H12 statistic calculated across windows of chromosome 2 for population cohorts from central Kenya. Peaks in H12 values are observed at the Cyp6aa/p gene cluster (2R:28,460,000-28,580,000), Keap1 (2R:40,926,195-40,945,169), Coeae2f (2 L:28,548,433-28,550,748) and Coeae2G-7G (2 L:37,282,152-37,298,223)

Supplementary Material 10: Supplementary Figure 10. Investigation of signals of selection with the H12 statistic calculated across windows of chromosome 2 for population cohorts from northwestern Kenya. A peak in H12 values was observed for the Cyp6aa/p gene cluster (2R:28,460,000-28,580,000)

Supplementary Material 11: Supplementary Figure 11. Investigation of signals of selection with the H12 statistic calculated across windows of chromosome 3 for population cohorts from central Kenya. No clear peaks in H12 values are observed

Supplementary Material 12: Supplementary Figure 12. Investigation of signals of selection with the H12 statistic calculated across windows of chromosome 3 for population cohorts from northwestern Kenya. No clear peaks in H12 values are observed

Supplementary Material 13.

Supplementary Material 14.

## Data Availability

The sequences of the samples identified in this study were submitted to the European Nucleotide Archive (ENA) (accession nos. ERR11784911-ERR12031958). The pipeline scripts used in this study are available at: https://malariagen.github.io/malariagen-data-python/latest/Ag3.html.

## Abbreviations

AChE: Acetylcholinesterase
AIMs: Ancestry informative marketers
BWA: Burrows–Wheeler Aligner
CBRD: Center for Biotechnology Research and Development
CNV: Copy Number Variations
DGK: Diacylglycerol kinase
DDT: dichloro-diphenyl-trichloroethane
GABA: γ-aminobutyric acid
GATK: Genome Analysis ToolKit
HMM: hidden Markov model
IRS: Indoor Residual Spray
KEMRI: Kenya Medical Research Institute
NMCP: National Malaria Control Programme
PAMCA: Pan-Africa Mosquito Control Association
PCA: Principal Component Analysis
PUBReC: Pwani University, Biosciences Research Centre
SHAPEIT: Segmented HAPlotype Estimation and Imputation Tools
s.l.: *sensu lato*
SNP: single nucleotide polymorphism
s.s.: *sensu stricto*
WHO: World Health Organization
WDL: Workflow Description Language
Vgsc: voltage-gated sodium channel
