# Supplementary figures and images for: Genomic surveillance reveals geographical heterogeneity and differences in known and novel insecticide resistance mechanisms in Anopheles arabiensis across Kenya
Source: BMC Genomics. 2025 Jul 1;26:599. doi: 10.1186/s12864-025-11788-3 (PMC12210584; doi:10.1186/s12864-025-11788-3)

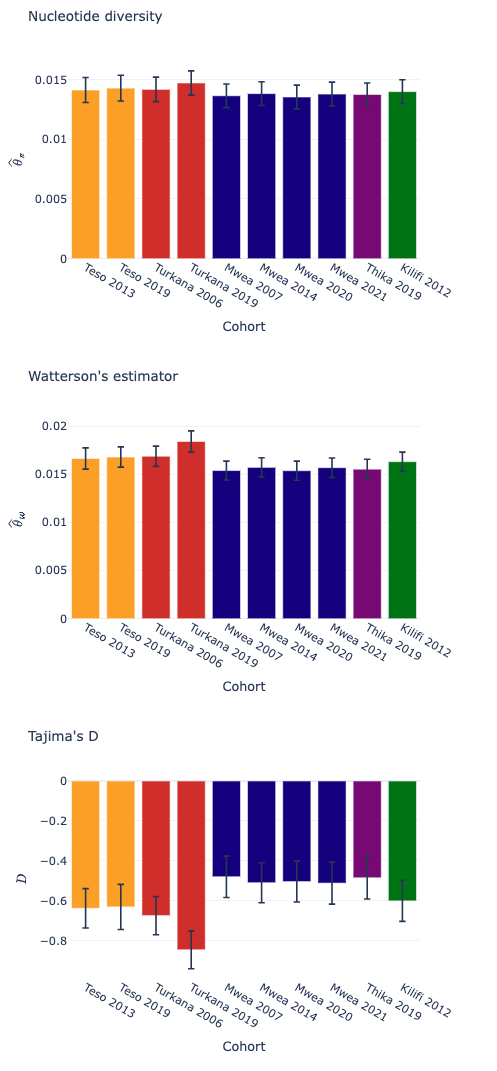

Supplement: Supplementary file 1 — Supplementary Material 1: Supplementary Figure 1. Bar plots of genetic diversity statistics for population cohorts, including nucleotide diversity (π), Watterson’s Theta (θ)and Tajima’s D. [file 12864_2025_11788_MOESM1_ESM.png]

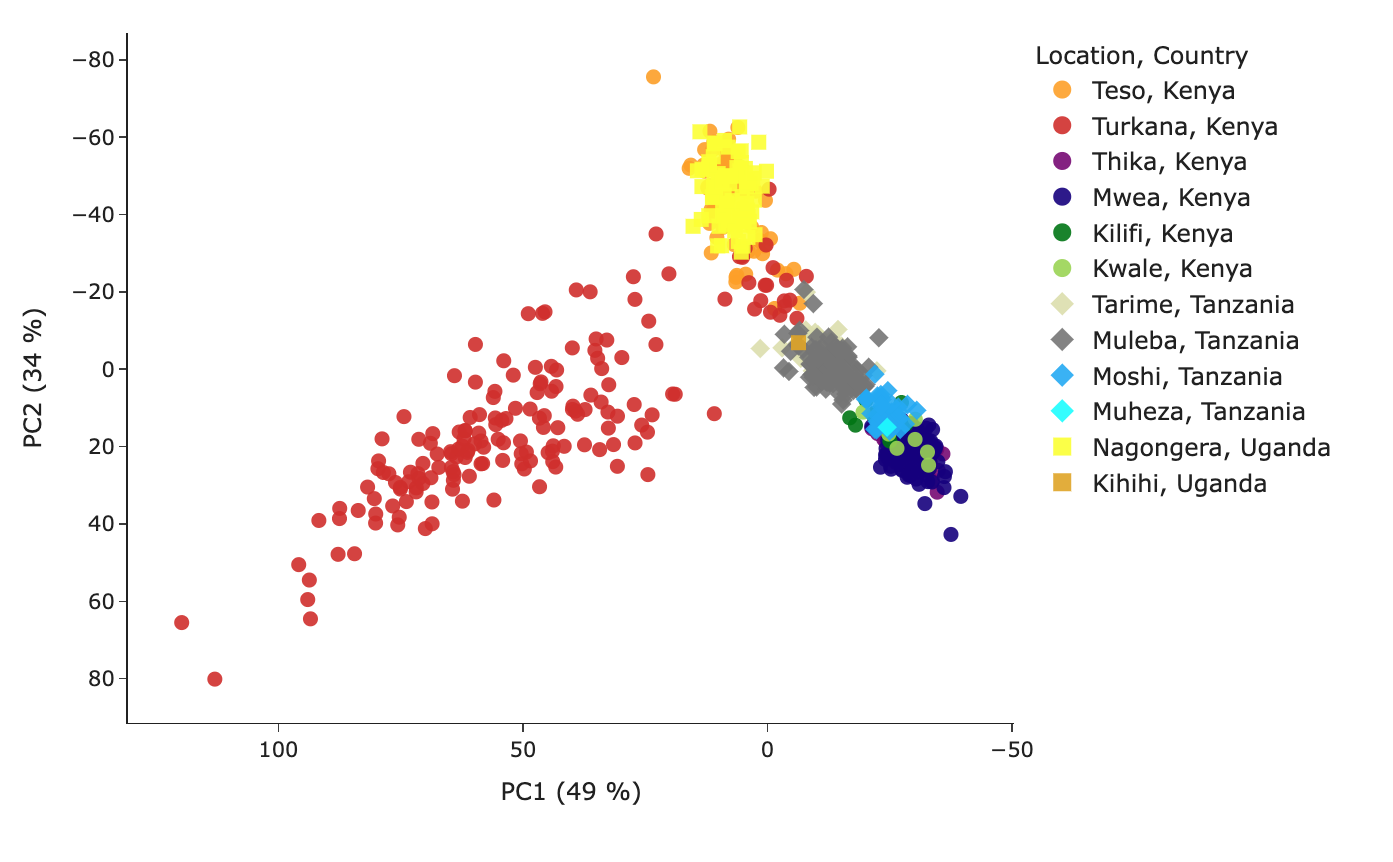

Supplement: Supplementary file 2 — Supplementary Material 2: Supplementary Figure 2. PCA to investigate the population structure of An. arabiensis from Kenya in relation to the neighbouring countries of Uganda and Tanzania. [file 12864_2025_11788_MOESM2_ESM.png]

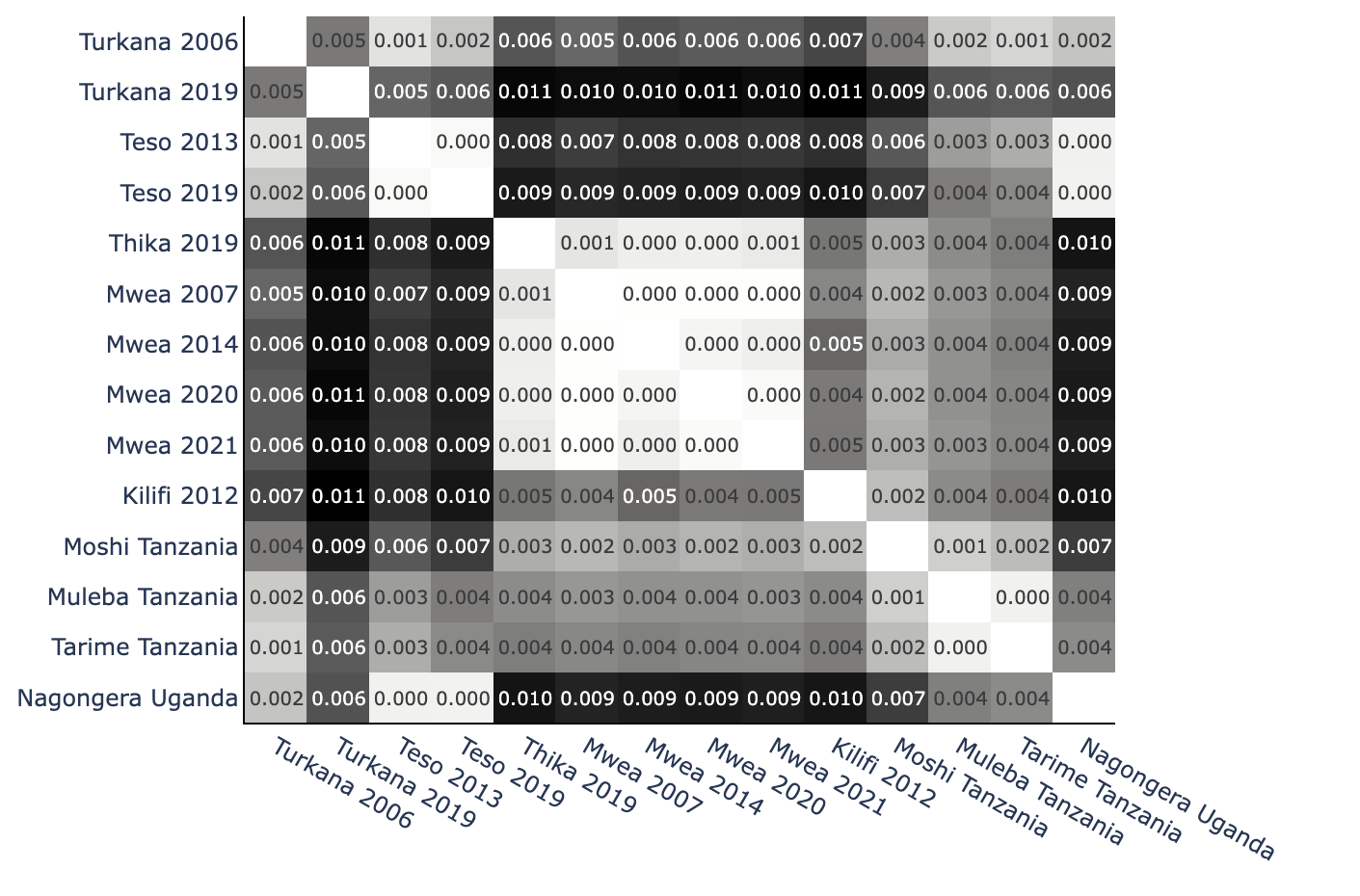

Supplement: Supplementary file 3 — Supplementary Material 3: Supplementary Figure 3. Investigation of the genetic differentiation of population cohorts with Hudson’s pairwise FST [file 12864_2025_11788_MOESM3_ESM.png]

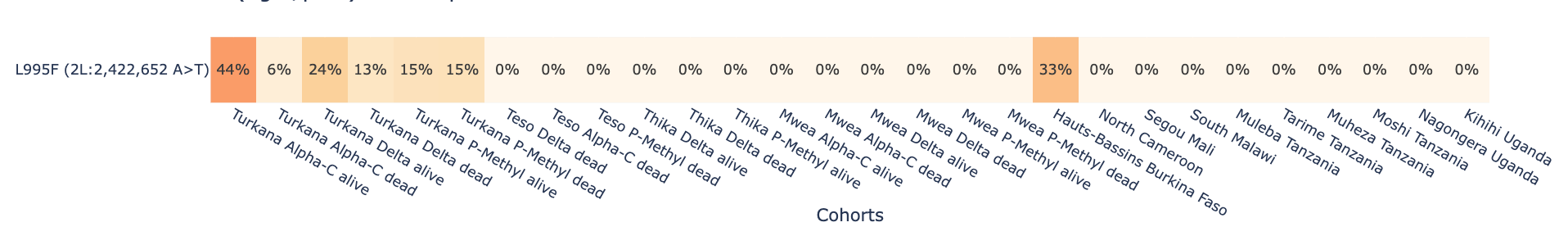

Supplement: Supplementary file 4 — Supplementary Material 4: Supplementary Figure 4. Investigation of the frequencies of the voltage-gated sodium channel substitution L995F in African An. coluzzii. [file 12864_2025_11788_MOESM4_ESM.png]

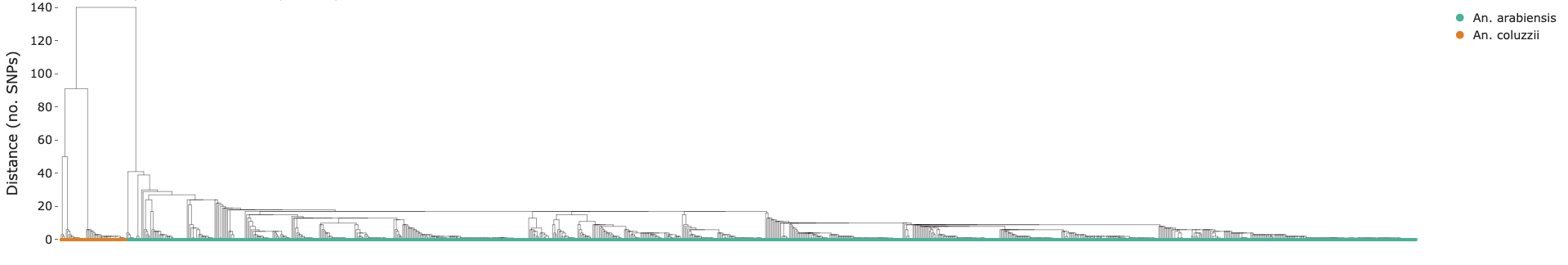

Supplement: Supplementary file 5 — Supplememtary Material 5: Supplementary Figure 5. Investigation of haplotype sharing with hierarchical clustering of the voltage-gated sodium channel gene [file 12864_2025_11788_MOESM5_ESM.png]

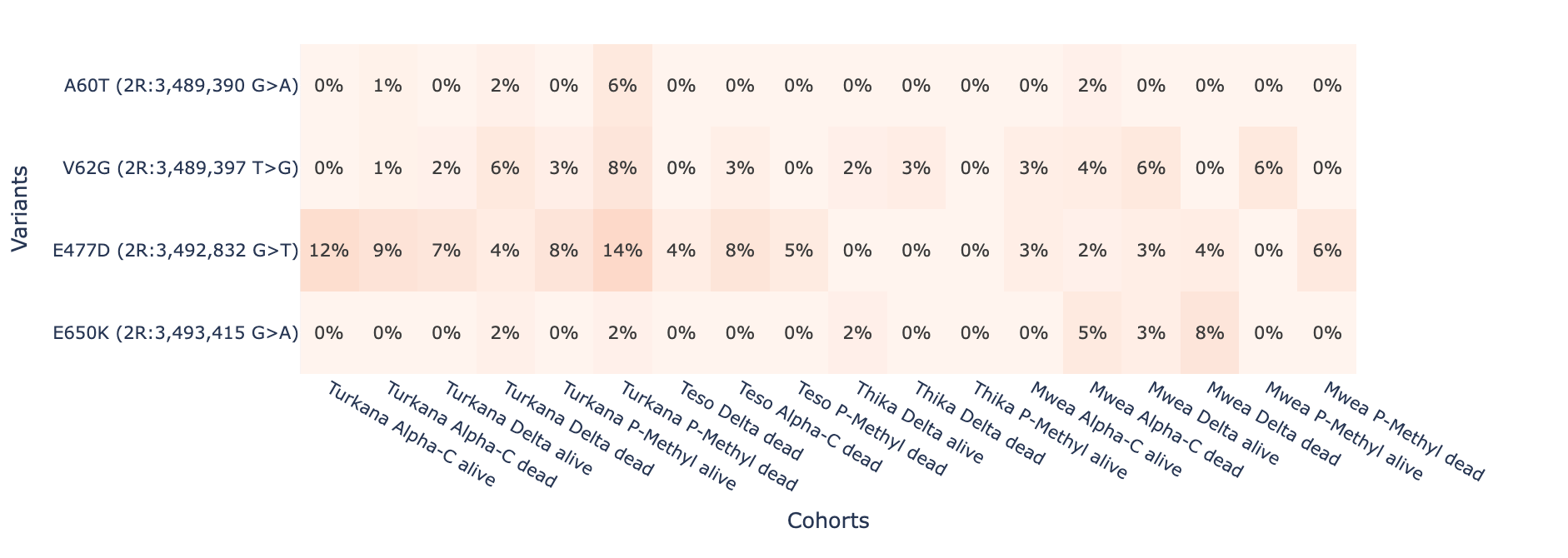

Supplement: Supplementary file 6 — Supplementary Material 6: Supplementary Figure 6. Investigation of amino acid substitutions at the ACE-1 locus [file 12864_2025_11788_MOESM6_ESM.png]

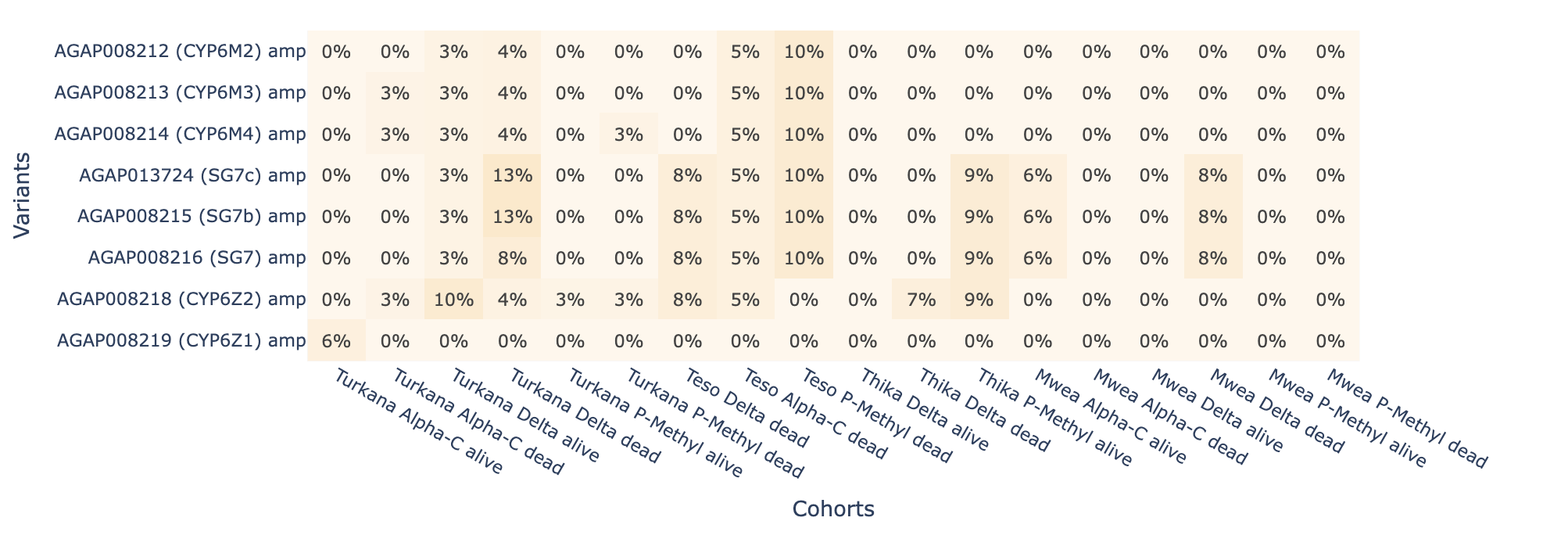

Supplement: Supplementary file 7 — Supplementary Material 7: Supplementary Figure 7. Investigation of CNV frequencies in the Cyp6z/m gene cluster [file 12864_2025_11788_MOESM7_ESM.png]

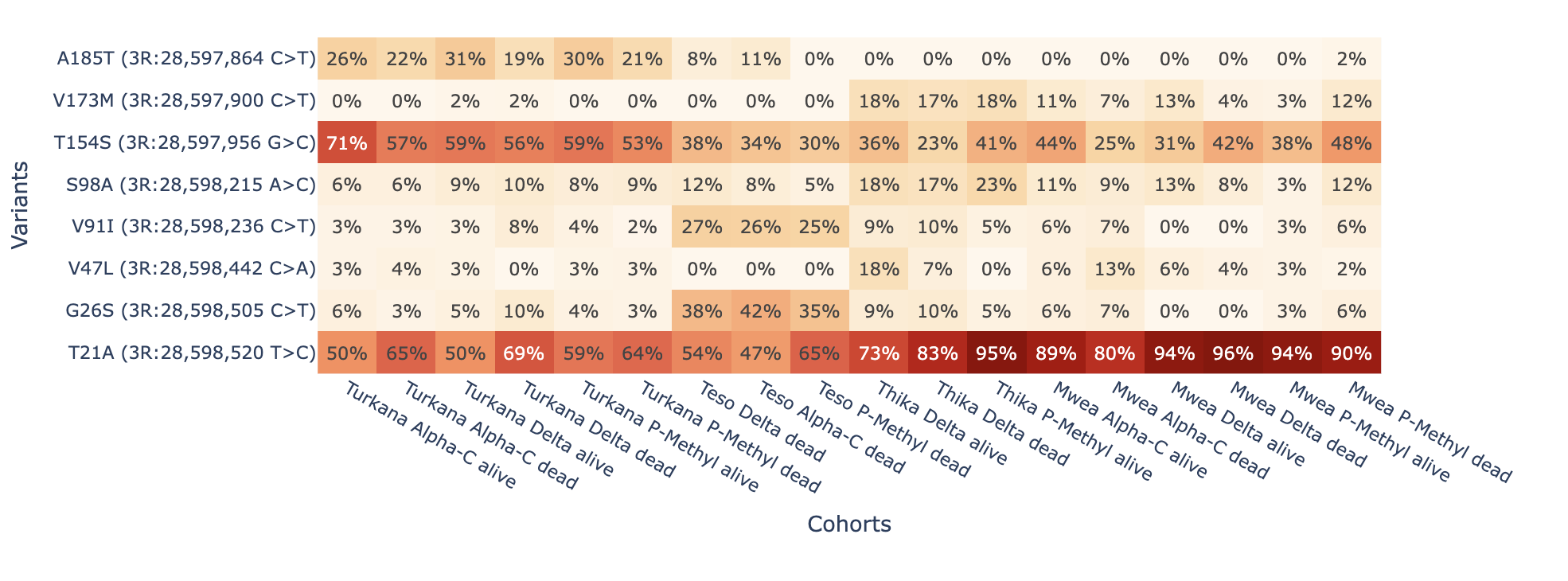

Supplement: Supplementary file 8 — Supplementary Material 8: Supplementary Figure 8. Investigation of amino acid substitutions at the GST locus [file 12864_2025_11788_MOESM8_ESM.png]

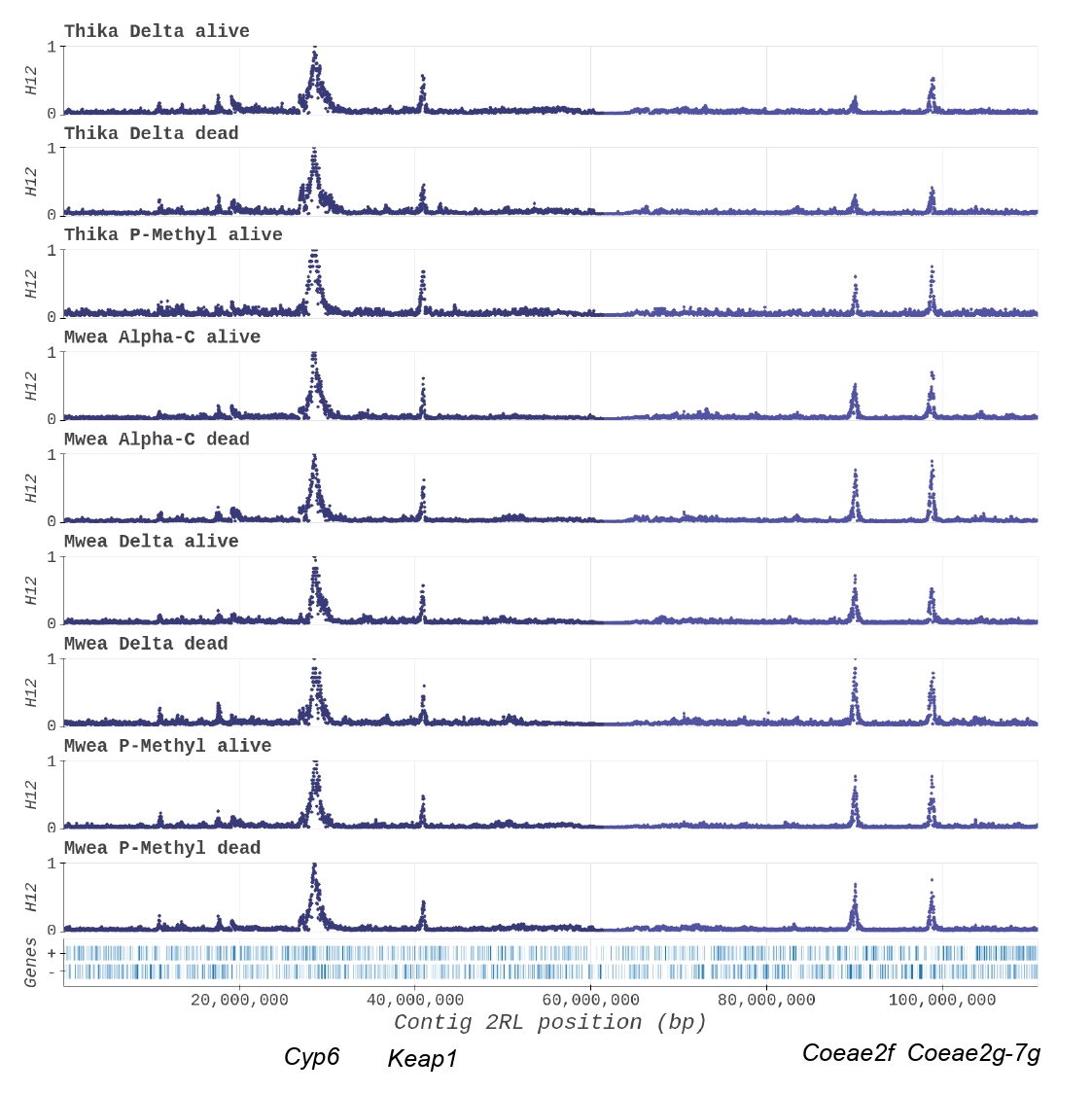

Supplement: Supplementary file 9 — Supplementary Material 9: Supplementary Figure 9. Investigation of signals of selection with the H12 statistic calculated across windows of chromosome 2 for population cohorts from central Kenya. Peaks in H12 values are observed at the Cyp6aa/p gene cluster (2R:28,460,000-28,580,000), Keap1 (2R:40,926,195-40,945,169), Coeae2f (2 L:28,548,433-28,550,748) and Coeae2G-7G (2 L:37,282,152-37,298,223) [file 12864_2025_11788_MOESM9_ESM.jpg]

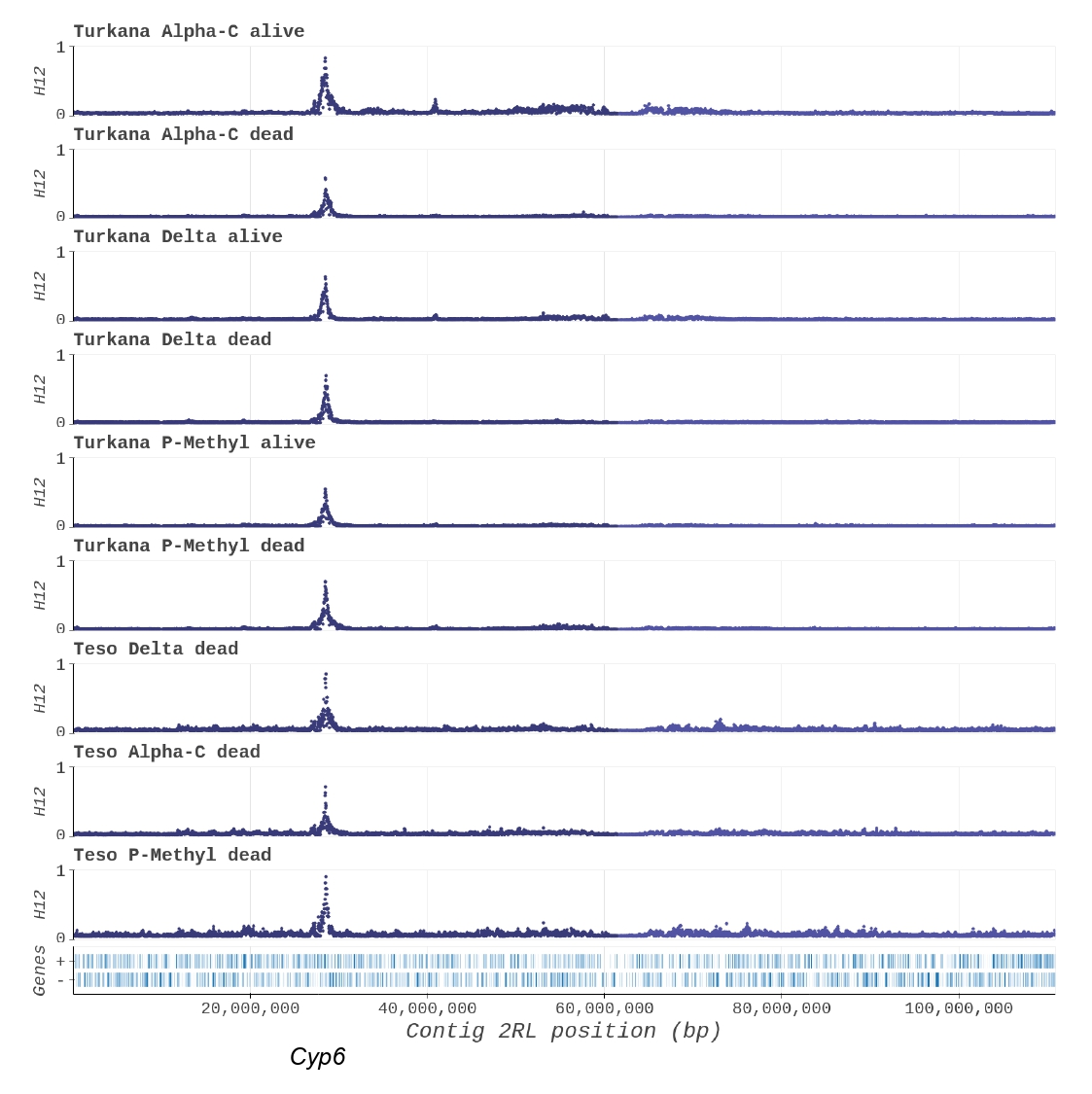

Supplement: Supplementary file 10 — Supplementary Material 10: Supplementary Figure 10. Investigation of signals of selection with the H12 statistic calculated across windows of chromosome 2 for population cohorts from northwestern Kenya. A peak in H12 values was observed for the Cyp6aa/p gene cluster (2R:28,460,000-28,580,000) [file 12864_2025_11788_MOESM10_ESM.jpg]

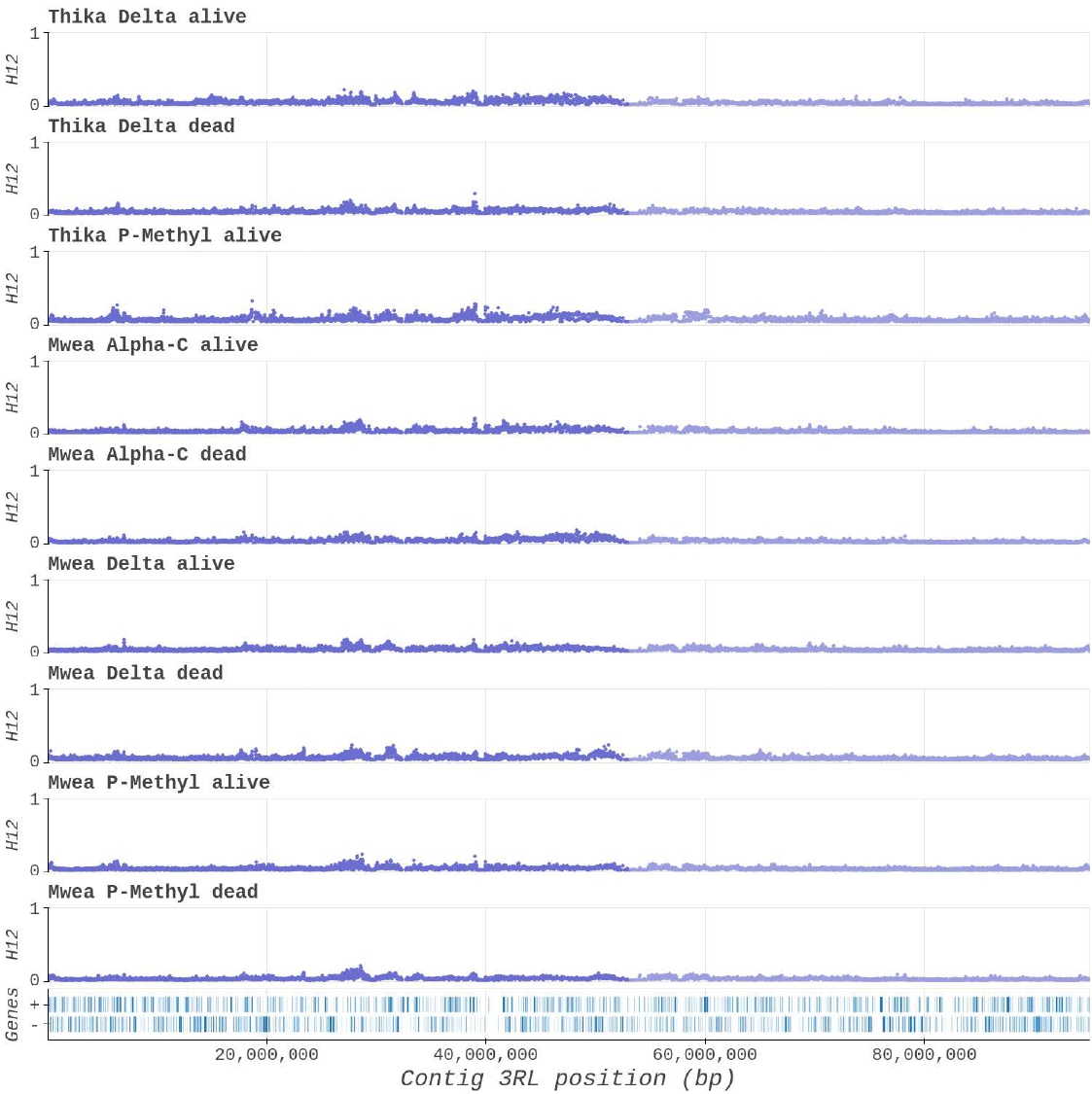

Supplement: Supplementary file 11 — Supplementary Material 11: Supplementary Figure 11. Investigation of signals of selection with the H12 statistic calculated across windows of chromosome 3 for population cohorts from central Kenya. No clear peaks in H12 values are observed [file 12864_2025_11788_MOESM11_ESM.jpg]

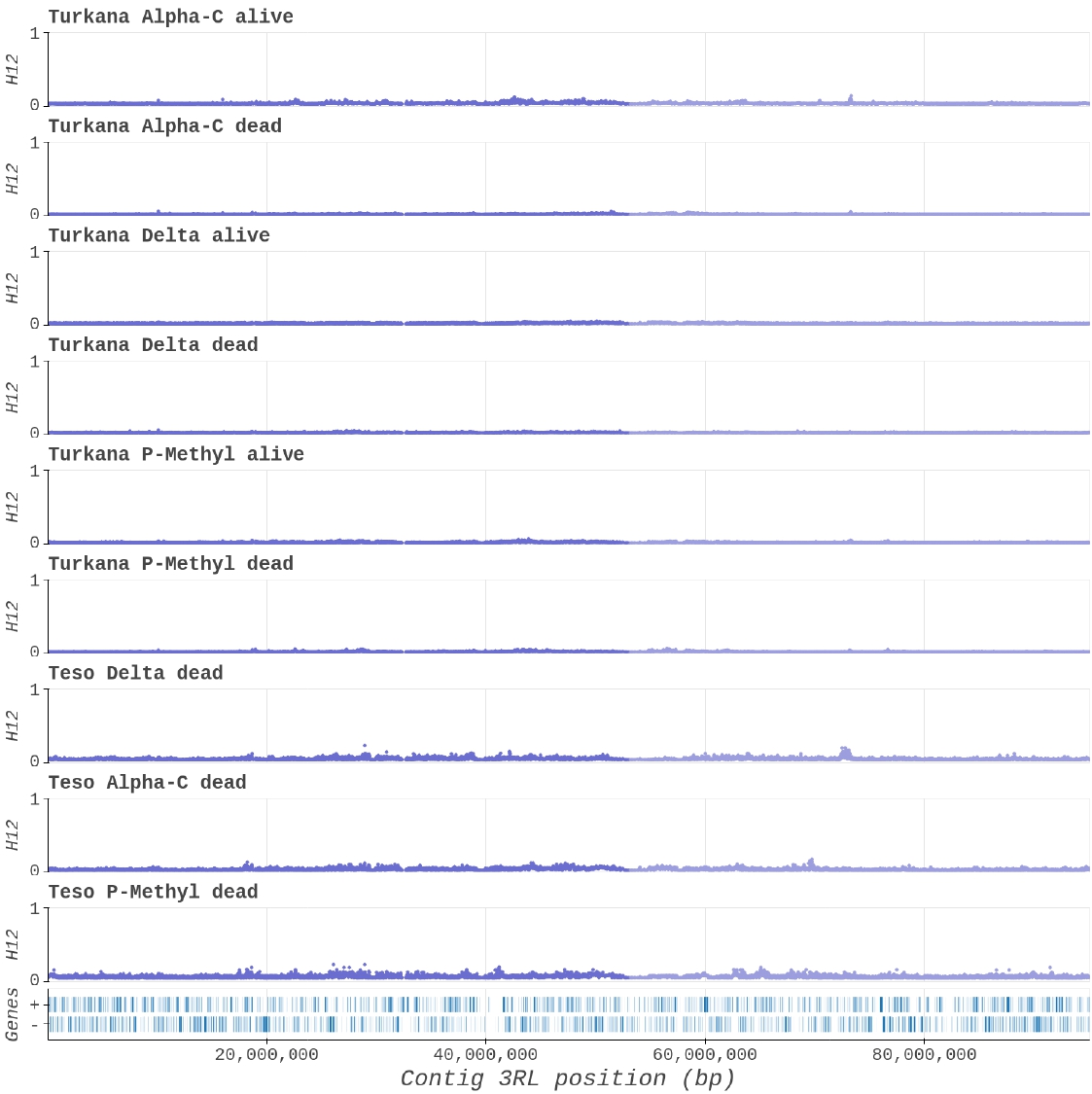

Supplement: Supplementary file 12 — Supplementary Material 12: Supplementary Figure 12. Investigation of signals of selection with the H12 statistic calculated across windows of chromosome 3 for population cohorts from northwestern Kenya. No clear peaks in H12 values are observed [file 12864_2025_11788_MOESM12_ESM.jpg]
